# Correction: Explaining predictive factors in patient pathways using autoencoders

**DOI:** 10.1371/journal.pone.0315113

**Published:** 2024-12-03

**Authors:** Hugo De Oliveira, Martin Prodel, Ludovic Lamarsalle, Vincent Augusto, Xiaolan Xie

The first and last names of second, third, fourth and fifth authors are incorrectly switched. The correct author names are: Martin Prodel, Ludovic Lamarsalle, Vincent Augusto, Xiaolan Xie. The correct citation is: De Oliveira H, Prodel M, Lamarsalle L, Augusto V, Xie X (2022) Explaining predictive factors in patient pathways using autoencoders. PLOS ONE 17(11): e0277135. https://doi.org/10.1371/journal.pone.0277135.
