# Supplementary material for: Explaining predictive factors in patient pathways using autoencoders
Source: PLoS One. 2022 Nov 10;17(11):e0277135. doi: 10.1371/journal.pone.0277135 (PMC9648714; doi:10.1371/journal.pone.0277135)
Supplement: S1 Appendix — The supplementary materials include details on the conducted experiments (hyperparameters, deep learning architectures), as well as all the quantitative and qualitative results obtained. (PDF) [file pone.0277135.s001.pdf]

Appendix

This appendix presents supplementary materials, including details on experiments, quantitative and qualitative results. Table A.1 details the hyperparameters tuning setting for ML methods. c is the number of features, and n the size of the training set. Figure A.1 details the architectures of DL models. Details of quantitative results are provided in Table A.2. Performances obtained on both train and test event logs are presented, highlighting a general overfitting for all methods. Table A.3 presents the performances for AE methods, using event logs with: (i) without hierarchy knowledge (only using codes as appearing in the initial event log), and (ii) with the hierarchy knowledge (as described in the methodology of Section IV. The percentage of improvement between (i) and (ii) is presented, showing that the use of the hierarchy knowledge slightly increases performances. Finally, Table A.4 shows performances obtained by using AE with recurrent encoder and decoder. These results shows that better performances can be obtained in comparison to the dense architecture used in the paper (Figure 4). However, explanations obtained were not satisfying, as no particular patterns were highlighted. As mentioned in the conclusion of this paper, investigations on these issues are part of the future work.

**model**	**parameter**	**values**
DT	max_depth	$[\![2, n]\!]$
	max_features	$[\![1, c]\!]$
	min_samples_leaf	$[\![2, n]\!]$
	class_weight	{None, balanced}
RF	n_estimators	500
	max_depth	$[\![2, n]\!]$
	max_features	$[\![1, c]\!]$
	min_samples_leaf	$[\![2, n]\!]$
	class_weight	{None, balanced}
LR	C	500
	class_weight	{None, balanced}
	max_iter	10000
LGBM	lambda_l1	$[1^{e-8}, 10.0]$
	lambda_l2	$[1^{e-8}, 10.0]$
	num_leaves	$[\![2, 256]\!]$
	feature_fraction	$[0.4, 1.0]$
	bagging_fraction	$[0.4, 1.0]$
	bagging_freq	$[\![1, 7]\!]$
	min_child_samples	$[\![5, 100]\!]$

TABLE A.1: Hyperparameters description for ML models tuning with Optuna.

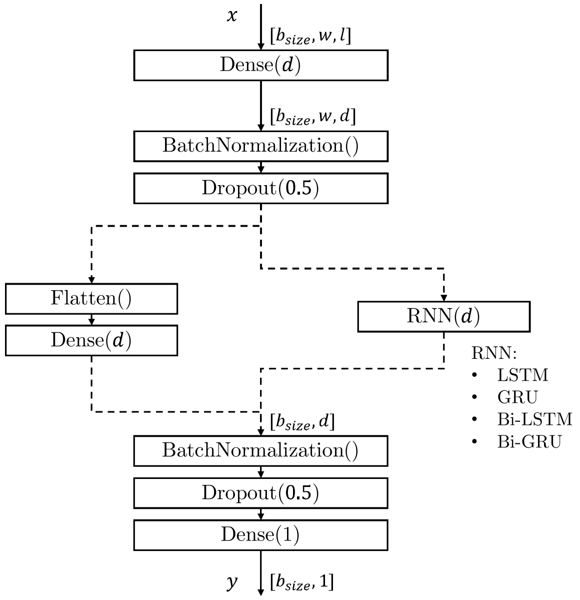

Fig. A.1: Schematic representation of deep learning architectures.

			**TRAIN**			**TEST**		
			AUC-ROC	AUC-PR	MCC	AUC-ROC	AUC-PR	MCC
ML	(LOF)	DT	0.726	0.641	0.316	0.698	0.597	0.262
		RF	0.761	0.684	0.382	0.727	0.640	0.320
		LR	0.756	0.678	0.378	0.739	0.656	0.352
		LGBM	0.948	0.931	0.779	0.746	0.666	0.369
	(TW)	DT	0.725	0.635	0.323	0.684	0.599	0.245
		RF	0.814	0.756	0.461	0.729	0.640	0.320
		LR	0.782	0.714	0.418	0.739	0.656	0.350
		LGBM	0.954	0.940	0.809	0.747	0.660	0.363
DL		Dense	0.852	0.806	0.538	0.736	0.653	0.352
		LSTM	0.811	0.755	0.461	0.752	0.681	0.367
		GRU	0.809	0.750	0.459	0.751	0.676	0.364
		BiLSTM	0.818	0.768	0.470	0.752	0.675	0.358
		BiGRU	0.803	0.746	0.448	0.754	0.675	0.364
AE	$\mathcal{J}_\theta^F$	Regular	0.813	0.777	0.488	0.724	0.622	0.321
		DAE	0.790	0.752	0.449	0.725	0.627	0.311
		VAE	0.788	0.747	0.449	0.722	0.629	0.305
	$\mathcal{J}_\theta^I$	Regular	0.773	0.693	0.413	0.746	0.667	0.359
		DAE	0.774	0.694	0.412	0.744	0.663	0.352
		VAE	0.777	0.697	0.415	0.746	0.669	0.368

TABLE A.2: Quantitative results for all methods, on train and test data.

		Without hierarchy			With hierarchy			Improvement (%)		
		AUC-ROC	AUC-PR	MCC	AUC-ROC	AUC-PR	MCC	AUC-ROC	AUC-PR	MCC
$\mathcal{J}_\theta^F$	Regular	0.720	0.631	0.313	0.724	0.622	0.321	0.569	-1.466	2.270
	DAE	0.716	0.621	0.304	0.725	0.627	0.311	1.316	0.945	2.173
	VAE	0.718	0.609	0.295	0.722	0.629	0.305	0.654	3.432	3.601
$\mathcal{J}_\theta^I$	Regular	0.739	0.658	0.354	0.746	0.667	0.359	0.957	1.259	1.271
	DAE	0.741	0.661	0.353	0.744	0.663	0.352	0.392	0.374	-0.118
	VAE	0.740	0.664	0.358	0.746	0.669	0.368	0.884	0.692	2.722

TABLE A.3: Comparison between the performances of AE methods obtained on the test set, depending on the use or not of the hierarchical knowledge.

			TEST results		
			AUC-ROC	AUC-PR	MCC
$\mathcal{J}_\theta^F$	Regular	Dense	0.724	0.622	0.321
		LSTM	0.710	0.620	0.294
		GRU	0.732	0.632	0.339
		BiLSTM	0.687	0.584	0.269
		BiGRU	0.729	0.634	0.314
	DAE	Dense	0.725	0.627	0.311
		LSTM	0.711	0.617	0.316
		GRU	0.734	0.634	0.329
		BiLSTM	0.726	0.629	0.327
		BiGRU	0.726	0.629	0.326
	VAE	Dense	0.722	0.629	0.305
		LSTM	0.718	0.637	0.326
		GRU	0.734	0.639	0.330
		BiLSTM	0.706	0.609	0.295
		BiGRU	0.728	0.634	0.320
$\mathcal{J}_\theta^I$	Regular	Dense	0.746	0.667	0.359
		LSTM	0.748	0.668	0.365
		GRU	0.748	0.670	0.372
		BiLSTM	0.754	0.676	0.369
		BiGRU	0.753	0.676	0.365
	DAE	Dense	0.744	0.663	0.352
		LSTM	0.750	0.672	0.366
		GRU	0.748	0.669	0.361
		BiLSTM	0.751	0.673	0.369
		BiGRU	0.753	0.677	0.363
	VAE	Dense	0.746	0.669	0.368
		LSTM	0.744	0.666	0.360
		GRU	0.748	0.672	0.358
		BiLSTM	0.746	0.667	0.357
		BiGRU	0.753	0.675	0.358

TABLE A.4: Quantitative results on the train set for AE methods with dense and recurrent architectures.

Finally, details on qualitative results are provided for both $\mathcal{J}_\theta^F$ and $\mathcal{J}_\theta^I$ (with VAE), including the t-SNE projections with y distributions (Figure A.2-A.3), visualization of mean elements $\bar{x}_0$, $\bar{x}_1$ and $\mathcal{E}$ (Figure A.4-A.5), with identified predictive factors (Figure A.6-A.7), and corresponding relative risks (Figure A.8-A.8). Regarding relative risks, $\mathcal{J}_\theta^F$ and $\mathcal{J}_\theta^I$ share some predictive factors identified. However, one can notice that while being characteristics, the values obtained from $\mathcal{J}_\theta^F$ are lower that the ones obtained by $\mathcal{J}_\theta^I$. This consideration aligns with the predictive performances of $\mathcal{J}_\theta^I$ being higher than the ones of $\mathcal{J}_\theta^F$.

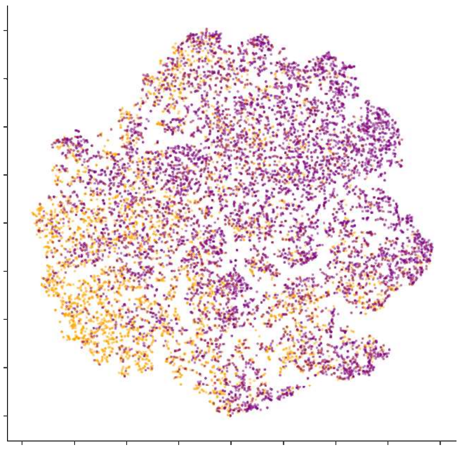

(a) t-SNE projection - Train

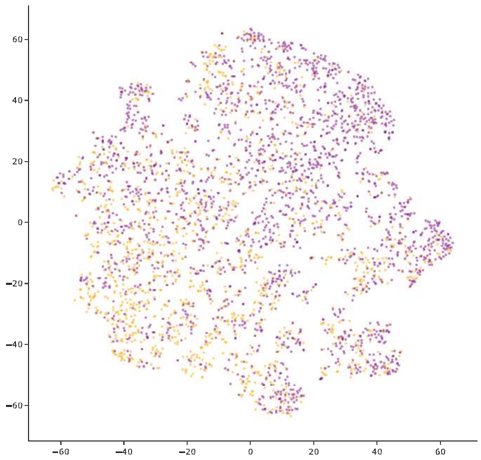

(b) t-SNE projection - Test

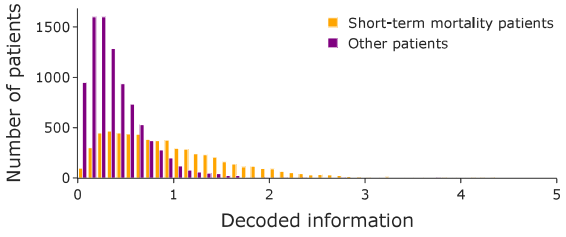

(c) y^F - Train

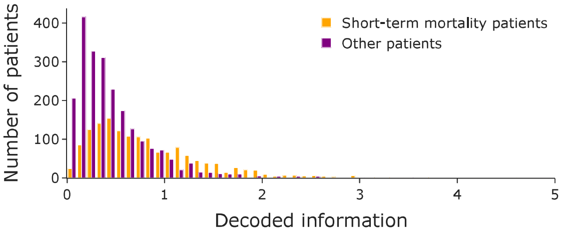

(d) y^F - Test

Fig. A.2: t-SNE projection of encoded patients and analysis of y^F distributions ($\mathcal{J}_\theta^F$ with a VAE architecture).

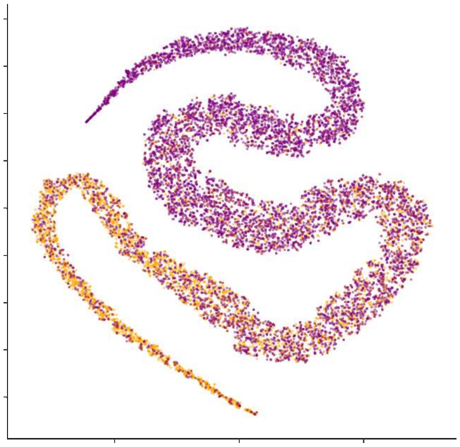

(a) t-SNE projection - Train

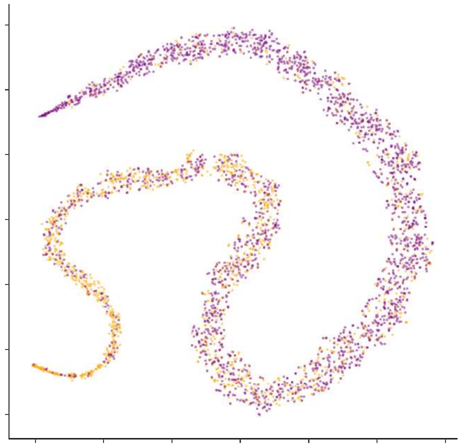

(b) t-SNE projection - Test

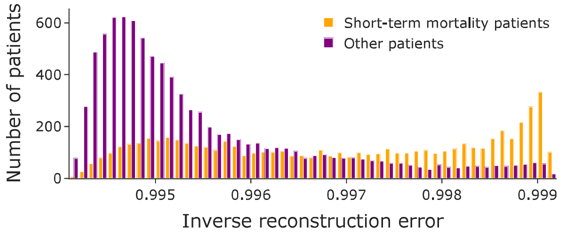

(c) y^I - Train

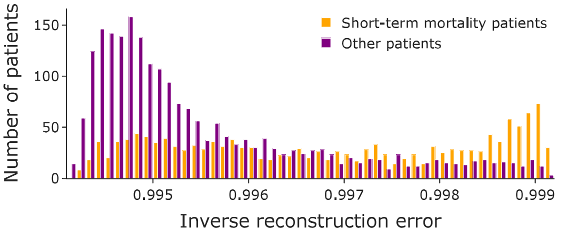

(d) y^I - Test

Fig. A.3: t-SNE projection of encoded patients and analysis of y^I distributions ($\mathcal{J}_\theta^I$ with a VAE architecture).

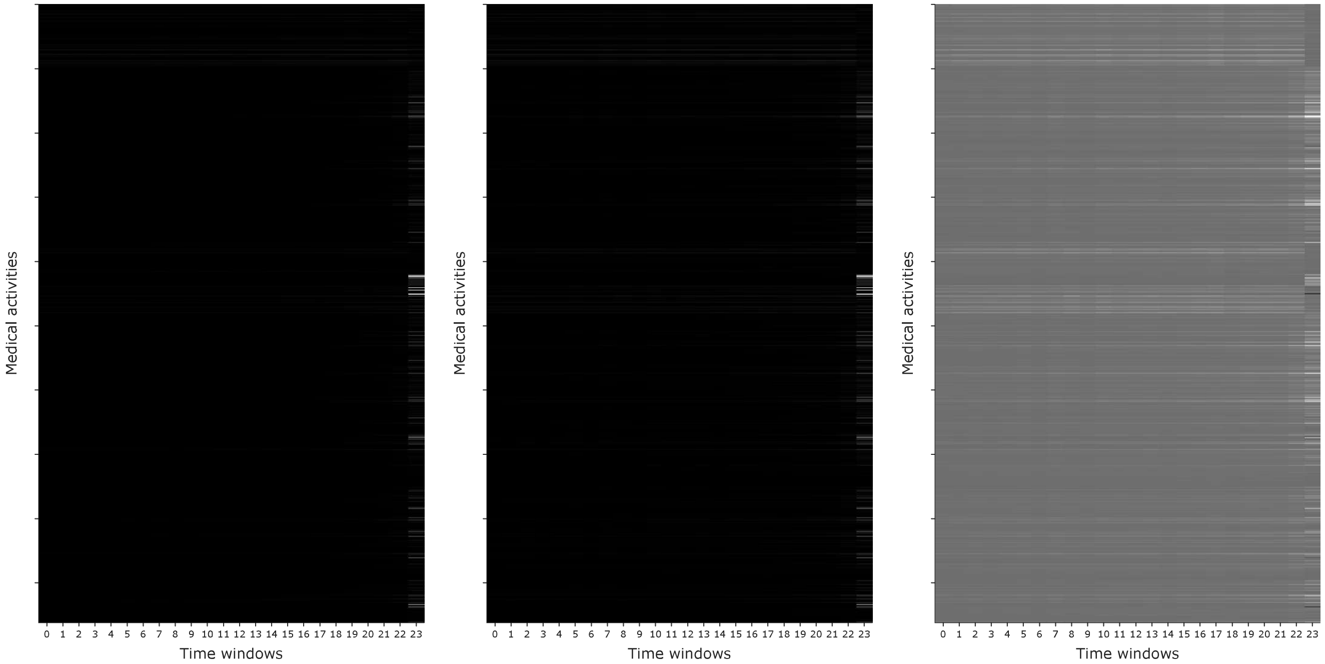

(a) $\bar{x}_0$ (b) $\bar{x}_1$ (c) $\mathcal{E} = \bar{x}_1 - \bar{x}_0$

Fig. A.4: Explanation using $\bar{x}_0$ and $\bar{x}_1$ to create $\mathcal{E}$ ($\mathcal{J}_\theta^F$ with a VAE architecture).

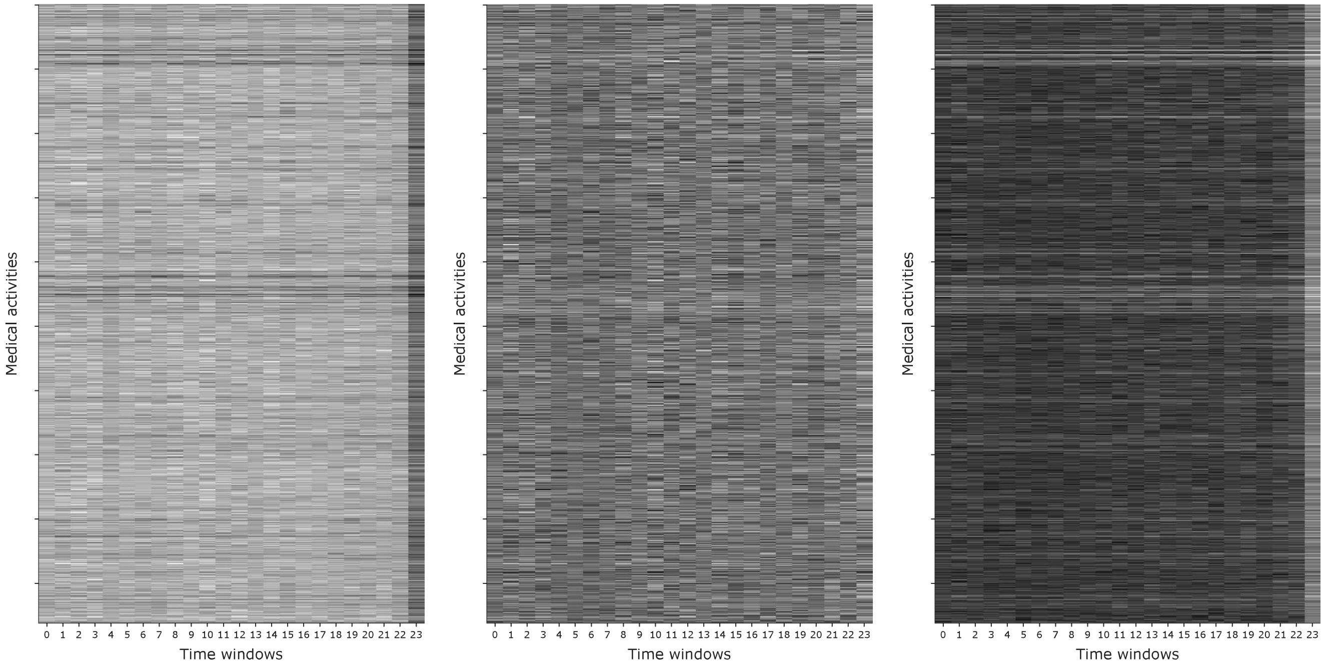

(a) $\bar{x}_0$ (b) $\bar{x}_1$ (c) $\mathcal{E} = \bar{x}_1 - \bar{x}_0$

Fig. A.5: Explanation using $\bar{x}_0$ and $\bar{x}_1$ to create $\mathcal{E}$ ($\mathcal{J}_\theta^I$ with a VAE architecture).

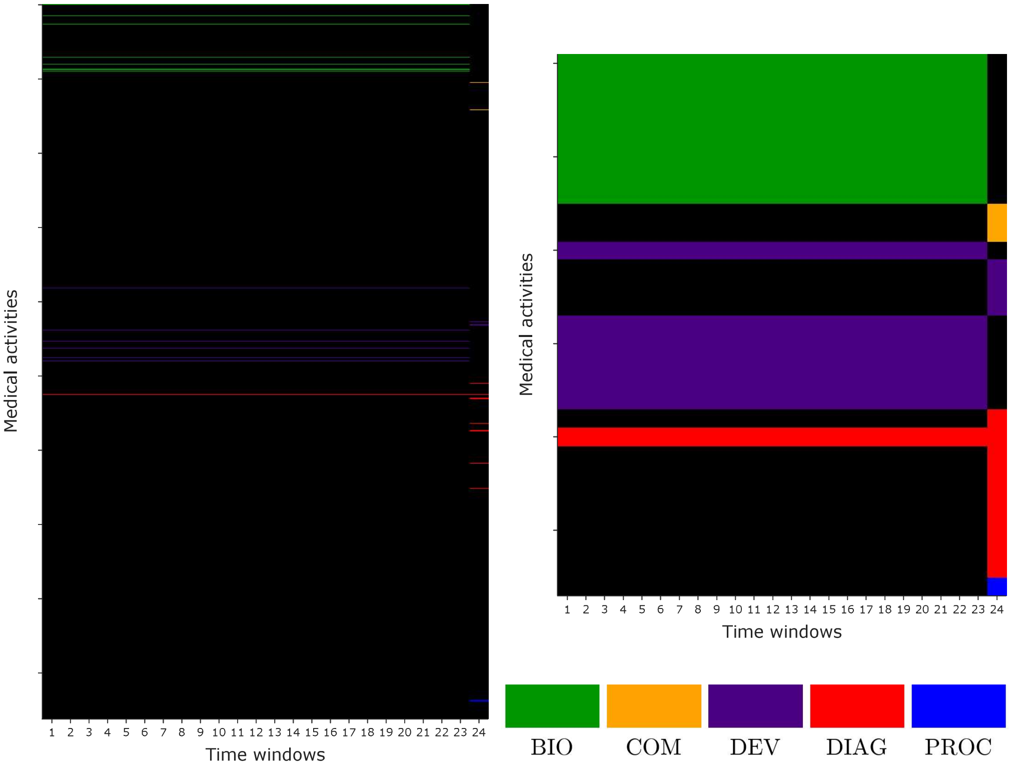

(a) $\mathcal{E}$ after selection of top activities

(b) Minimal representation of $\mathcal{E}$

Fig. A.6: Identification of predictive factors in $\mathcal{E}$ ($\mathcal{J}_\theta^F$ with a VAE architecture).

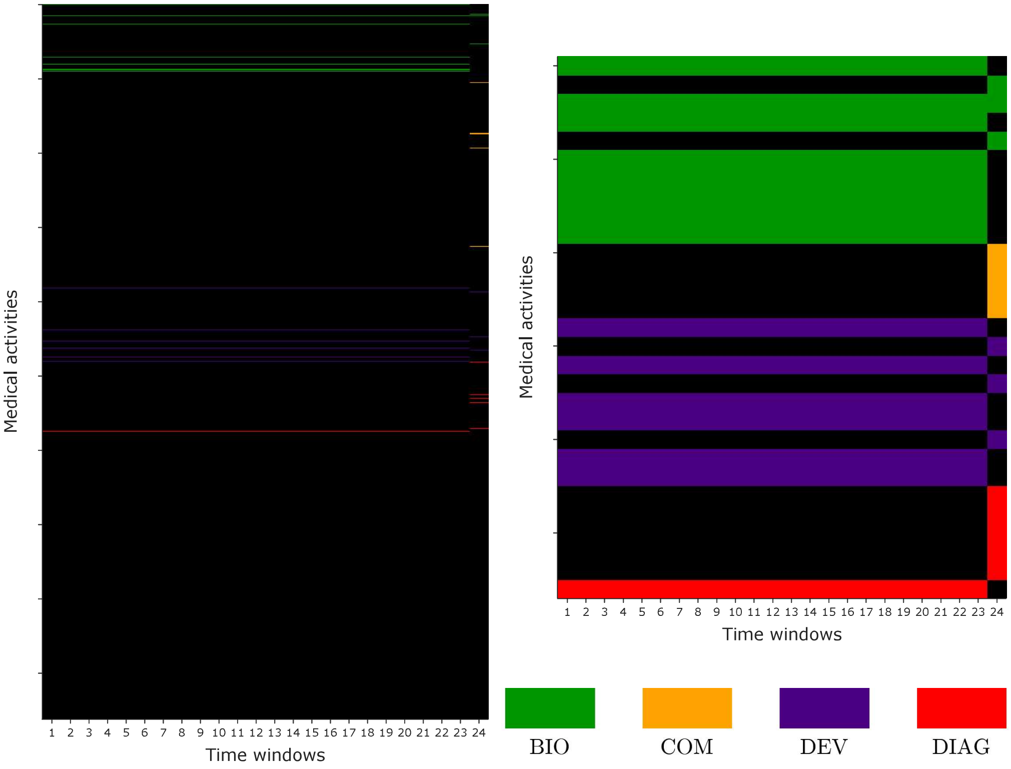

(a) $\mathcal{E}$ after selection of top activities

(b) Minimal representation of $\mathcal{E}$

Fig. A.7: Identification of predictive factors in $\mathcal{E}$ ($\mathcal{J}_\theta^I$ with a VAE architecture).

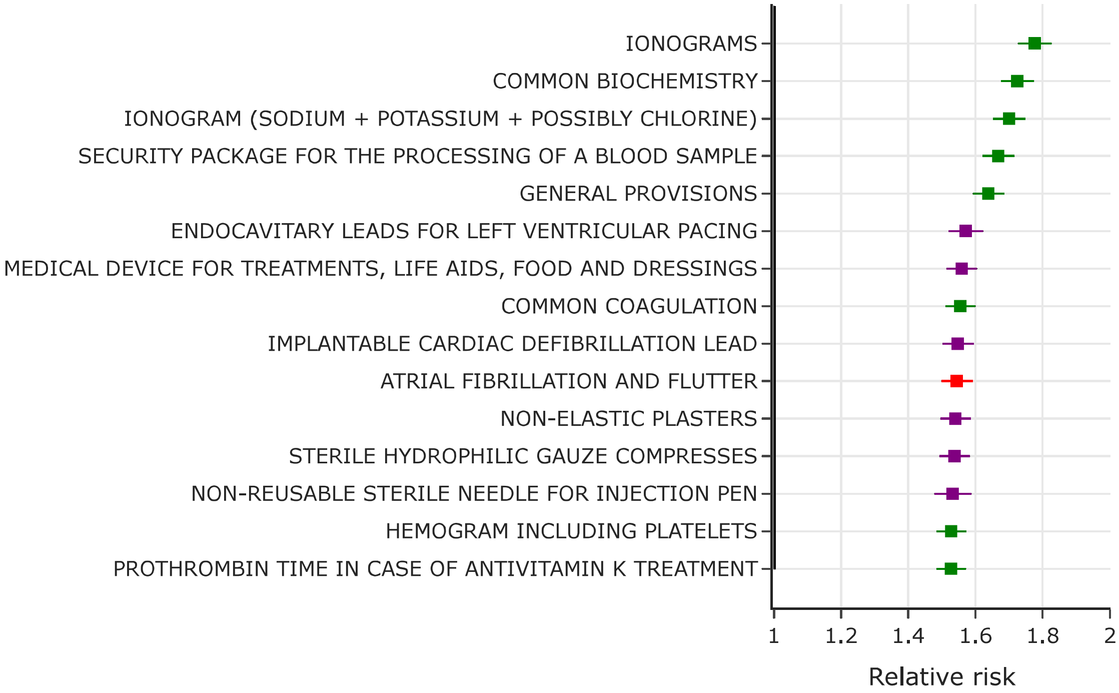

(a) Relative risks deduced from $\mathcal{E}$ (frequent events)

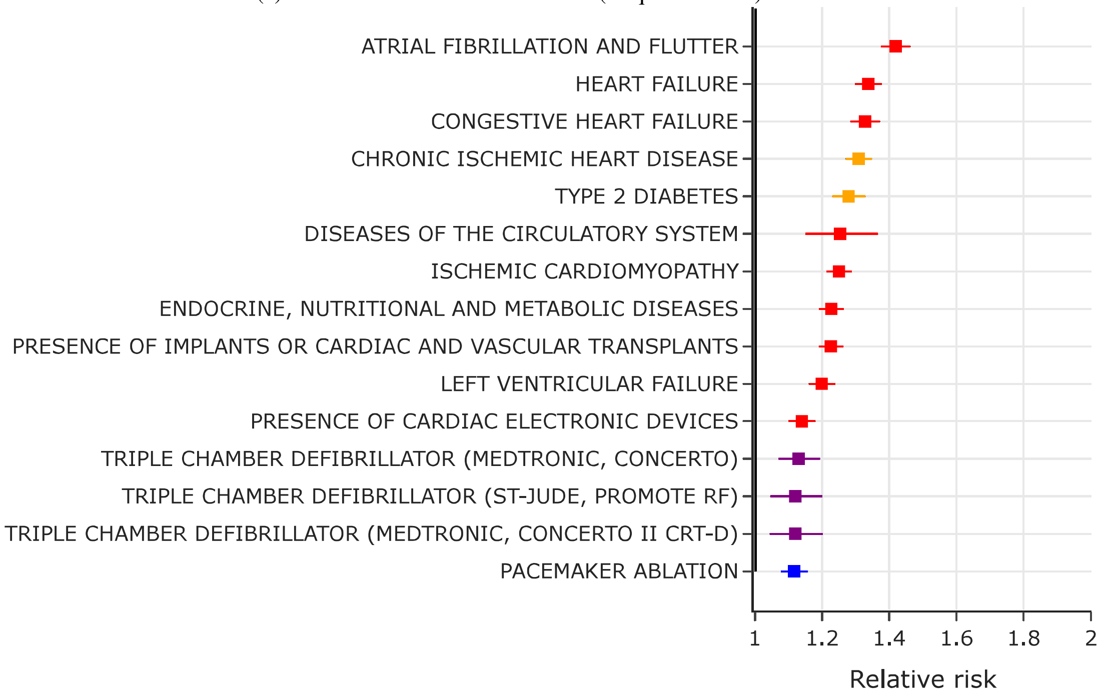

(b) Relative risks deduced from $\mathcal{E}$ (last time window)

Fig. A.8: Relative risks ($\mathcal{J}_\theta^F$ with a VAE architecture).

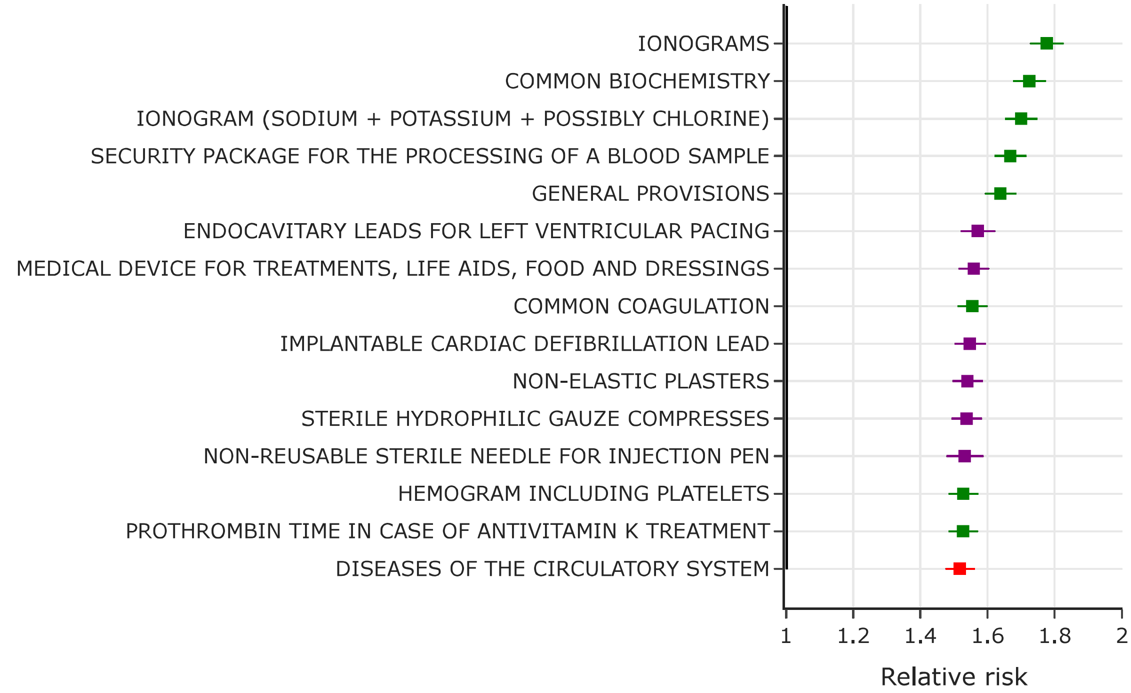

(a) Relative risks deduced from $\mathcal{E}$ (frequent events)

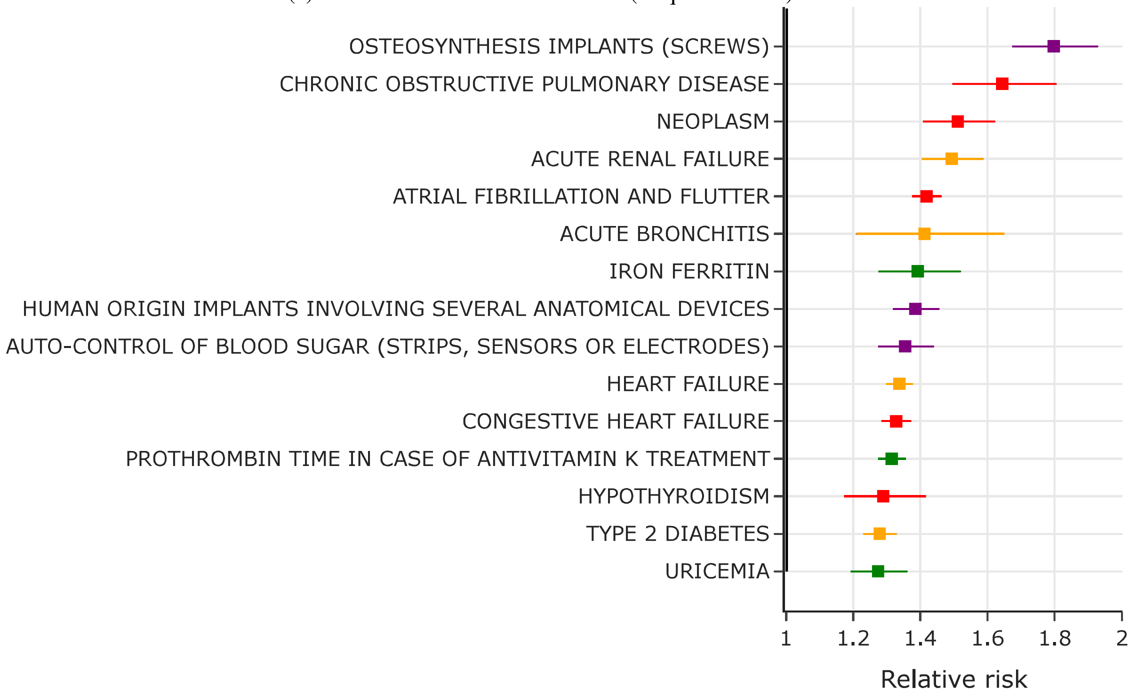

(b) Relative risks deduced from $\mathcal{E}$ (last time window)

Fig. A.9: Relative risks ($\mathcal{J}_\theta^I$ with a VAE architecture).
