# Supplementary material for: Explaining predictive factors in patient pathways using autoencoders
Source: PLoS One. 2022 Nov 10;17(11):e0277135. doi: 10.1371/journal.pone.0277135 (PMC9648714; doi:10.1371/journal.pone.0277135)
Supplement: Attachment — Submitted filename: Response to Reviewers.pdf [file pone.0277135.s002.pdf]

Manuscript title: Explaining predictive factors in patient pathways using autoencoders

ID: PONE-D-22-13428

Subject: Revision submission

Dear reviewers,

Thank you very much for the interesting comments and useful suggestions provided regarding the manuscript entitled “Explaining predictive factors in patient pathways using autoencoders”.

By following your recommendations, a new version of the manuscript has been submitted for publication. Majority of your comments have been addressed and your suggestions have been included in this new version. In order to facilitate the review, a point-by-point answer to your comments is presented below.

Therefore, we truly hope that changes applied will satisfy all the requirements, making our paper suitable for publication in PLOS ONE.

Sincerely,

Hugo De Oliveira, on behalf of Martin Prodel, Ludovic Lamarsalle, Vincent Augusto and Xiaolan Xie.

Reviewer #1

"In this article titled "Explaining predictive factors in patient pathways using autoencoders", the authors investigated the performance of autoencoders to explain predictive factors in patient pathways. To develop the above, the authors develop a method to transform and represent complex medical event logs. The developed autoencoders have been compared with other deep learning and machine learning methods by testing on retrospective data from the SNIIRAM database, and demonstrates competitive prediction performances. The use of autoencoders for explaining predictive factors in patient pathways is an interesting application. However, the document can be improved and made clearer to the audience. The grammar of the manuscript needs to be improved and the methodology-result section needs some rearrangement. In addition, discussion on certain aspects of the results is lacking."

Thank you for highlighting great opportunities for us to improve the manuscript, and for the very detailed suggestions. Please find bellow a point-by-point answer to all the recommendations.

"1) Section 3 – line 202 (below eqn 8): The meaning of the sentence " The explanation element…that the any input elements…" is not clear due to apparent mistake in sentence structure. Consider rewriting."

The structure of the sentence has been addressed.

"2) Section 5.2.1 – line 322: The acronym AUC-ROC was not defined, but was only defined in a later Section 5.2.3 (line 360)."

Definition has been added.

"3) Section 5.2.2: The authors state the selection of activation functions, layer dimension and loss function. Any particular reason why these hyperparameters were chosen? Was there any analysis/ hyperparameter tuning being made to optimise these hyperparameters, particularly because they are the same for all five DL models?"

It is true that no hyperparameter tunning has been done to select the architecture of deep learning models. The activation function has been chosen as state-of-the-art choice for binary classification. Layer dimensions have been selected to have similar model size to compare with the encoder. Regarding activation function, hyperbolic tangent has been selected as a widespread choice for recurrent networks.

"4) Section 5.2.3 – line 348: acronym ELBO is not defined"

Acronym is now defined.

"5) Section 5.2.3 - line 365: "DT, RF and LR…. [43]. Deep learning… [44]". These sentences are general statements (not specific to the autoencoder section). It is suggested that a separate section (5.2.4) be added to include these statements as to provide clarity and avoid confusion to the readers. The last

sentence of Section 5.2.3 "The experiments…. Windows 10 OS" could also be shifted to this newly added section."

Section 5.2.4 added (Computational details).

"6) Section 5.3 and Fig 5: The authors present quantitative results, including values for the AUC-ROC, AUC-PR, and MCC. The reviewer suggests that the authors give a simple explanation of these results, particularly what these values represent in relation to the case study presented? Also, any particular reason of the discrepancy in results between AE(J_I) and AE(J_F)? This should also be included in the discussion."

A description has been added in Section 5.3 in order to comment the difference obtained between AE_JI vs AE_JF.

"7) Section 5.4: The authors use "(a)" and "(b)" to reference to Fig 6. It is suggested that the authors use "Fig 6 (a)" and "Fig 6 (b)" as to improve clarity and avoid confusion. furthermore, "(c)" is mentioned in line 414, however such figure does not exist in Fig. 6. The reviewer believes this refers to Fig 7 instead, please amend."

References to figures have been changed accordingly.

"8) Section 5.4 – line 416,417: The authors mention that "the relative risks with a 95% confidence interval….are presented in (a) and (b), respectively". Again, this should be in reference to Fig 8 but was not mentioned. Please amend."

References to figures have been changed accordingly.

"9) Section 5.4: the authors presented a method of validating the assumptions made from the results of Fig 6a by computing the relative risks. The following text describing this methodology should be exclusive to the methods section, rather than the results/discussion."

The methodology presented here is in fact a specific methodology based on the results obtained for that particular case study (i.e. the obtention of two categories of patterns – frequent and last time window – with for each pattern, a measure of the relative risk). The section has been reorganized in order to better highlight the specificity of the described methodology to that particular case study.

"10) Conclusion – line 456: The authors mentioned that better strategies can be used to improve prediction performance and explainability. Could the authors provide some possible examples/potential methods to achieve this? If not, this sentence remains highly speculative."

This is indeed too speculative, investigations are still part of future work. The sentence has been rephrased.

"11) This study focuses exclusively on patient pathways to explain predictive factors, while omitting patient characteristics such as sex, age, and race. Would the inclusion of these characteristics in the

study improve performance and/or uncover hidden patterns? How would the omission of these variables affect potential clinical applications? As the mortality or other patient outcomes could be affected by such factors , ie there is an inherent predisposition of the patient due to these factors."

This was only slightly addressed in the conclusion. More details have been provided about how to extend the methodology to include patient characteristics.

"1) Section 3 – line 157: "…input data x and return a lower…". Should be "returns"
2) Section 4 – line 221: "..the considered case study". Missing fullstop.
3) Section 4 – line 225: "..all level of…". Should be "all levels of"
4) Section 5.2.1 – line 322: "…, evaluation the mean…" should be "…and evaluating the mean…"
5) Section 5.2.2 – line 330: "The 4 other architecture replace…" should be "The 4 other architectures replace…". Also, suggested to use spelling for numbers that are less than 10, ie "four" instead of "4".
6) Section 5.2.3 – line 346: "The training process consists in…". Should it be "consists of"?
7) Section 5.2.3 – line 354: "For DL ans AE…". Typo of the word "and"
8) Section 5.3 – line 373 "…, were the proposed methods…" should be "…, where the proposed methods…".'
9) Section 5.4 – line 431: "…that various level…" should be "…that various levels"
10) Section 5.4 – line 393: "…train data by computing…" should be "…training data by computing…"
11) Conclusion – line 460: "..of the time widow". Typo of the word "window""

Thank you for providing such detailed changes to apply in the manuscript. All the changes have been addressed.

Reviewer #2

"The abstract:
After reading the whole paper, I found that the abstract lacks the jism of the work. The work done is not reflected in the abstract. I would advise to review same. I understand that there is a limitation on the number of words for the abstract- However, it lacks the main aspect of the work."

The abstract has been enriched in order to better highlight the contributions while respecting the limitations.

"Introduction:
Provided the research gap and the challenges that need to be addressed.
The main contribution is to explain the causal factors and to devise a framework to model the system
Introduction is explicit and well- written
Literature review
Right information to understand the topic

Section 3:
Section 3 focuses on autoencoders- However, this section seems to be disjoint over the whole paper. The explanations with the formula are good. However, it would be better if this section was linked with the work conducted in this paper and not just a preliminary where a reader does not have the interest why this is being discussed here."

A subsection entitled \`Motivations\` has been added to Section 3, with the objective of better introducing this section of the paper (which is key for a technical description of the method), while better positioning the section with regard to the case study and the overall objective of the paper.

"Section 5:
Presents the case study. From what is reported is that a satisfactory amount of data was captured from 18, 678 patients
The authors have used machine learning (ML), deep learning (DL) and autoencoders (AE) to select and represent data. There are not enough details regarding deep learning.
Explainability of the parameters provided- However, the authors have not compared it with other author's works. I understand that it is different, since it shows the explainability part- However, there is a need to provide a discussion in relation with other work."

The choice has been made by the authors to focus on detailing the autoencoder methodologies because it was the main contribution of the paper. Details of all the deep learning architectures can be found in Supplementary material. Regarding comparison with other author's work, we tried to include the positioning of this work within the literature in Section 2. Regarding explainability, it is true that no comparison of qualitative results obtained from the proposed method to other explainable methods have been done. This has been added as a limitation in Section 6.

"One of the contributions of this paper is to validate the predictive factors extracted through relative risks, widely used in bio-statistical analysis. Some missing discussions in relation to the results and other people's work."

Relative risks have been used in the literature to measure the risk of an outcome between two groups. In general, the creation of these two groups is inherent to the study. In the context of our case study, the groups have been provided by the explanation of the method. To the best of our knowledge, this kind of analysis where the groups are suggested by the predictive model and where we observe a significant risk for one group versus the other have not been presented so far.

“Otherwise, it is a good piece of work.”
